# Matrix metalloproteases and TIMPs as prognostic biomarkers in breast cancer patients treated with radiotherapy: A pilot study

**DOI:** 10.1111/jcmm.14671

**Published:** 2019-09-30

**Authors:** María Auxiliadora Olivares‐Urbano, Carmen Griñán‐Lisón, Mercedes Zurita, Rosario del Moral, Sandra Ríos‐Arrabal, Francisco Artacho‐Cordón, Juan Pedro Arrebola, Amanda Rocío González, Josefa León, Juan Antonio Marchal, María Isabel Núñez

**Affiliations:** ^1^ Department of Radiology and Physical Medicine School of Medicine University of Granada Granada Spain; ^2^ Biopathology and Regenerative Medicine Institute (IBIMER) Centre for Biomedical Research University of Granada Granada Spain; ^3^ Department of Human Anatomy and Embryology School of Medicine University of Granada Granada Spain; ^4^ Department of Radiation Oncology Virgen de las Nieves University Hospital Granada Spain; ^5^ Biosanitary Research Institute, ibs.Granada Granada Spain; ^6^ Department of Preventive Medicine and Public Health School of Medicine University of Granada Granada Spain; ^7^ Bio‐Health Research Foundation of Eastern Andalusia ‐ Alejandro Otero (FIBAO) Granada Spain

**Keywords:** breast cancer patients, matrix metalloproteases, prognostic and predictive biomarkers, radiotherapy, tissue inhibitors

## Abstract

Breast cancer (BC) is the most common tumour in women and one of the most important causes of cancer death worldwide. Radiation therapy (RT) is widely used for BC treatment. Some proteins have been identified as prognostic factors for BC (Ki67, p53, E‐cadherin, HER2). In the last years, it has been shown that variations in the expression of MMPs and TIMPs may contribute to the development of BC. The aim of this pilot work was to study the effects of RT on different MMPs (‐1, ‐2, ‐3, ‐7, ‐8, ‐9, ‐10, ‐12 and ‐13) and TIMPs (‐1 to ‐4), as well as their relationship with other variables related to patient characteristics and tumour biology. A group of 20 BC patients treated with RT were recruited. MMP and TIMP serum levels were analysed by immunoassay before, during and after RT. Our pilot study showed a slight increase in the levels of most MMP and TIMP with RT. However, RT produced a significantly decrease in TIMP‐1 and TIMP‐3 levels. Significant correlations were found between MMP‐3 and TIMP‐4 levels, and some of the variables studied related to patient characteristics and tumour biology. Moreover, MMP‐9 and TIMP‐3 levels could be predictive of RT toxicity. For this reason, MMP‐3, MMP‐9, TIMP‐3 and TIMP‐4 could be used as potential prognostic and predictive biomarkers for BC patients treated with RT.

## 1 INTRODUCTION

Breast cancer (BC) is the most common tumour in women and the fifth cause of cancer death worldwide.^1^ BC is a heterogeneous disease at the inter‐ and intra‐tumour level, which is relevant to the prognosis and therapy of the disease.^2^ The type of BC, its location and other factors (differentiation grade, size, presence of different proteins—Ki67, p53, E‐cadherin—sentinel lymph node, patient age and response to treatment) are also relevant to the prognosis.^3^

Breast cancer can be classified according to location (in situ and invasive or infiltrating),^3^ histology (ductal, lobular, nipple and not otherwise specified),^4^ and presence or absence of oestrogen receptors (ER), progesterone receptors (PR) and human epidermal growth factor receptor‐2 (HER2). According to these receptors, BC is classified as luminal A or B (ER and PR positive), basal or triple negative (all negative) and HER2‐enriched (only HER2 positive) 5; being the triple negative, the subtype associated with worse outcome.^6^

Differentiation grade indicates the rate of tumour growth and dissemination, and it is determined based on how similar tumour cells compared with normal cells in breast tissue. Tumours can be differentiated into three grades: grade I or well differentiated; grade II or moderately differentiated; and grade III or poorly differentiated.^3^ Different proteins are considered markers of prognosis. There is evidence that Ki67 is involved in cell division,^7^ and its immunohistochemical (IHC) detection is used to evaluate tumour proliferation.^8^ The p53 protein is involved in cancer development.^9^ Several studies in mice have shown that p53 mutations can result in a more aggressive tumour behaviour and metastasis.^10^, ^11^ E‐cadherin mediates cell‐cell adhesion and is expressed by epithelial cells.^12^ Damage in E‐cadherin structure or alteration in its expression are related to tumour progression and metastasis.^13^

Involvement of the sentinel lymph node is not only important for disease prognosis but also for tumour invasion and metastasis into the lymph nodes.^14^ Sentinel lymph node biopsy is now essential to evaluate the local‐regional extension of the disease. Different studies have shown that patients with a negative sentinel lymph node biopsy do not require axillary lymph node dissection.^15^ Age and menopausal status are another factor to consider in BC development. Breast lobules undergo age‐related lobular involution (ARLI) which begins around the age of 40 and accelerates after menopause. ARLI is associated with a lower risk of BC.^16^, ^17^

Radiation therapy (RT) is used in the treatment of most tumours. RT regimen is different depending on the patient characteristics, threshold dose and the tissues and organs where tumour is located. In addition, RT can be combined with surgery and/or systemic therapy.^18^ In BC, the most commonly used RT regimens are conventional RT (45‐50 Gy in fractions of 2 Gy) and hypofractionated RT (normally 42.5 Gy in fractions of 2.66 Gy).^19^

The efficacy obtained with RT can vary according to the chosen RT regimen and to increased radioresistance in patients. RT toxicity may also appear and depends on the chosen RT regimen, total dose received, volume of irradiated breast and patient age. Skin toxicity is the most common adverse effect in BC patients and they can be acute (erythema, desquamation, ulceration and haemorrhage) or late/chronic (hyperpigmentation and telangiectasia). De Felice et al found that higher irradiated breast volume and conventional RT have a negative effect on acute skin toxicity.^18^

Matrix metalloproteases (MMPs) are a family of enzymes that differ in their structure, substrate specificity, sequence homology, cellular localization and secretion. For this reason, they are divided into different subfamilies: collagenases, gelatinases, matrilysins, stromelysins, membrane type MMPs and others MMPs.^20^, ^21^ MMPs are mainly involved in extracellular matrix (ECM) remodelling,^20^ but they are also capable of processing proteins unrelated to the ECM and activating other MMPs and proteases.^22^ MMPs are key regulators of cell‐cell interactions and perform different functions in a variety of normal biological 23 and carcinogenic processes (tumour growth, angiogenesis, degradation of collagen in basal membrane, changes in the epithelial‐mesenchymal transition [EMT], invasion and metastasis).^24^ These processes may be favored by the increase in MMP activity after RT. The balance between MMPs and theirs tissue inhibitors (TIMPs) playing a crucial role in cancer progression and metastasis.^25^

Endogenous TIMPs are not only endogenous inhibitors of MMPs, but they also have biological activities that are independent of MMPs including cell growth and differentiation, angiogenesis, apoptosis and synaptic plasticity.^26^ The four TIMPs described in humans (TIMP‐1 to ‐4) have different inhibition spectrum and affinity for human MMPs.^27^

Changes in MMP and TIMP expression may contribute to the development of BC, and these genes have been examined as potential prognostic serum biomarkers in BC.^24^ Different studies have linked high serum levels of MMPs and TIMPs with a poor prognosis 26, 28; specifically, they have been identified as predictors of adverse outcome and with poor survival.^29^, ^30^ Prognostic implications are related to cell type that expresses MMPs (stromal versus tumour cells).^24^ Several studies showed that MMP‐1, ‐7, ‐9, ‐11, ‐13 and ‐14 immunostaining of tumour cells, stromal fibroblasts and mononuclear inflammatory cells were associated with shorter relapse‐free survival.^31^, ^32^

Considering that RT could influence MMP and TIMP gene expression levels, this therapy could be used to interfere with the different steps of the metastatic cascade. Metastasis is the main cause of death in patients with cancer, and it has been estimated that approximately 90% of BC deaths arise from the metastatic spread of primary tumours.^33^ For this reason, the main objective of this work was to study the effects of RT on MMP and TIMP expression, as well as their relationship with other variables related to patient and tumour characteristics, and examine their role as prognostic and predictive factors in BC in relation to their key role in tumour invasion and metastasis.

## 2 MATERIALS AND METHODS

### 2.1 Recruitment and characteristics of patients

This pilot study was carried out in 20 patients with BC from the Virgen de las Nieves University Hospital in Granada. These patients were treated with either hypofractionated RT (16 sessions, 2.65 Gy/session) or conventional RT (25 sessions, 2 Gy/session). Three blood samples were collected from each patient at different times of the treatment, with a total of 60 samples. The first sample was taken approximately one week before starting RT; the second sample was taken during the RT (8 days for hypofractionated regimen and 11 days for conventional regimen, after the start of the treatment); and the third sample was taken at RT termination, usually on the last day of treatment. Measurement of protein levels was done after the patients had received approximately similar doses; therefore, it is unlikely that the fractionation regimen might have affected the serum levels of the proteins investigated. For both regimens, before RT patients had received 0 Gy; during RT, they received 21.2 Gy in the hypofractionated therapy and 22 Gy in conventional fractionated therapy. After RT, 42.4 and 50 Gy were administered for hypofractionated and conventional therapy, respectively. Considering that the α/β ratio for breast cancer is 3, the estimate of the biologically effective dose (BED) was 102.3 Gy and the equivalent dose (EQD 2) was 61.4 Gy for hypofractionated therapy. As for conventional fractionated therapy, BED was 104.1 Gy and EQD 2, 62.5 Gy. This reveals that dose differences according to the regimen used are minimal at the end of the treatment and they should not affect the results.

Samples were kept until analysis by the Biobank of the Public Health System of Andalusia in Granada. This study was approved by the corresponding ethical committee associated with grants PI‐730 and PIE16‐00045. Written informed consent was obtained from all the patients involved in this study. Patients were classified according to the different variables studied, including patient‐dependent variables (age and menopausal status), tumour biology‐dependent variables (classification based on hormones, differentiation grade, positive or negative E‐cadherin and p53, Ki67 percentage and sentinel lymph node involvement) and RT‐related variables (RT regimen, lymph node RT, presence of radiotoxicity). Table 1 describes in more detail the variables studied, the population (n) and the percentage compared with total population. The 6‐month BC recurrence is also shown.

**Table 1:** Description of the variables studied related to patient characteristics, tumour biology and RT (n = 20)

Variables	n	(%)	Recurrence	
			No	Yes
Age				
≤50 y	10	50	9	1
>50 y	10	50	8	2
Menopausal status				
Pre‐menopausal	10	50	9	1
Menopausal	6	30	5	1
Post‐menopausal	4	20	3	1
Type of carcinoma				
Invasive ductal	19	95	16	3
Invasive lobular	1	5	1	0
Tumour classification (ER, PR, HER2)				
Hormone‐negative	2	10	1	1
Hormone‐positive	18	90	16	2
Differentiation grade				
Grade I	9	45	8	1
Grade II	7	35	6	1
Grade III	4	20	3	1
E‐cadherin				
Positive	16	80	14	2
Negative	4	20	3	1
p53				
Positive	3	15	2	1
Negative	17	85	15	2
Ki67				
<20%	15	75	13	2
≥20%	5	25	4	1
Sentinel lymph node				
Yes	12	60	11	1
No	8	40	6	2
RT regimen				
Conventional	7	35	6	1
Hypofractionated	13	65	11	2
Lymph node RT				
Yes	9	45	7	2
No	11	55	10	1
RT toxicity				
No	2	10	2	0
Hyperpigmentation	1	5	1	0
Erythema	12	60	10	2
Radiodermitis	5	25	4	1
Chemotherapy				
Yes	11	55	9	2
No	9	45	8	1
Recurrence				
Healthy	17	85		
Sick	3	15		

### 2.2 Determination of MMP and TIMP serum levels by immunoassay

Bio‐Plex Data Pro software was used to determine and quantify the levels of the selected MMPs and TIMPs (MMP‐1, ‐2, ‐3, ‐7, ‐8, ‐9, ‐10, ‐12, ‐13, TIMP‐1, ‐2, ‐3 and ‐4), according to the protocols provided by BioRad in the Bio‐Plex Pro Human MMP and TIMP Assays kits (#171AM001M and #171AM002M). The normalization was realized according to the protocols, reconstituting the standards and controls from the kit and preparing the standard dilution series. The samples used were serum aliquots obtained from the stored blood samples. The Bio‐Plex Pro assays are immunoassays formatted on magnetic beads that use a principle similar to ELISA. Briefly, the capture antibodies are covalently coupled to the beads, and the formed complexes react with the sample containing the target biomarker. After a series of washes to remove unbound protein, a biotinylated detection antibody is added to create a sandwich complex. The final detection complex is formed with the addition of streptavidin‐phycoerythrin (SA‐PE) conjugate. PE serves as a fluorescent indicator. Data from the reactions are presented as median fluorescence intensity (MFI) as well as concentration of analyte (pg/ml) bound to each bead, which is proportional to the MFI of the fluorescent indicator signal.

### 2.3 GEO database

Studies with a similar aim were searched in different databases (cBioPortal, GDC Data Portal, GEO DataSets). We have not found any study that correlated the serum levels of MMPs and TIMPs with RT. Only one study that correlates MMPs and TIMPs with RT was found on the GEO database (GSE101920).^34^ This study shows the gene expression profile in BC biopsies taken both prior to RT, and after RT and radical mastectomy. Due to the limited number of matched samples (n = 5) obtained, the study was continued by focusing on the analysis of pre‐RT biopsies only.

### 2.4 Statistical analysis

Our results were expressed as median ± standard deviation (SD). The statistical analysis was performed with the IBM SPSS Statistics software package, *P* values < .05 were considered significant. The values considered significant were named differently, depending on the value of *P*: *(*P* < .05) and **(*P* < .01). The non‐parametric tests of Kruskal‐Wallis and U‐Mann‐Whitney were used for producing the histograms and the non‐parametric Spearman's Rho test for the correlation matrices. GraphPad Prism 8.0.0 and R Statistical Computing Environment 3.4.0 software were used for graphing the data sets.

## 3 RESULTS

### 3.1 Time course of MMP and TIMP serum levels

**Figure 1:**
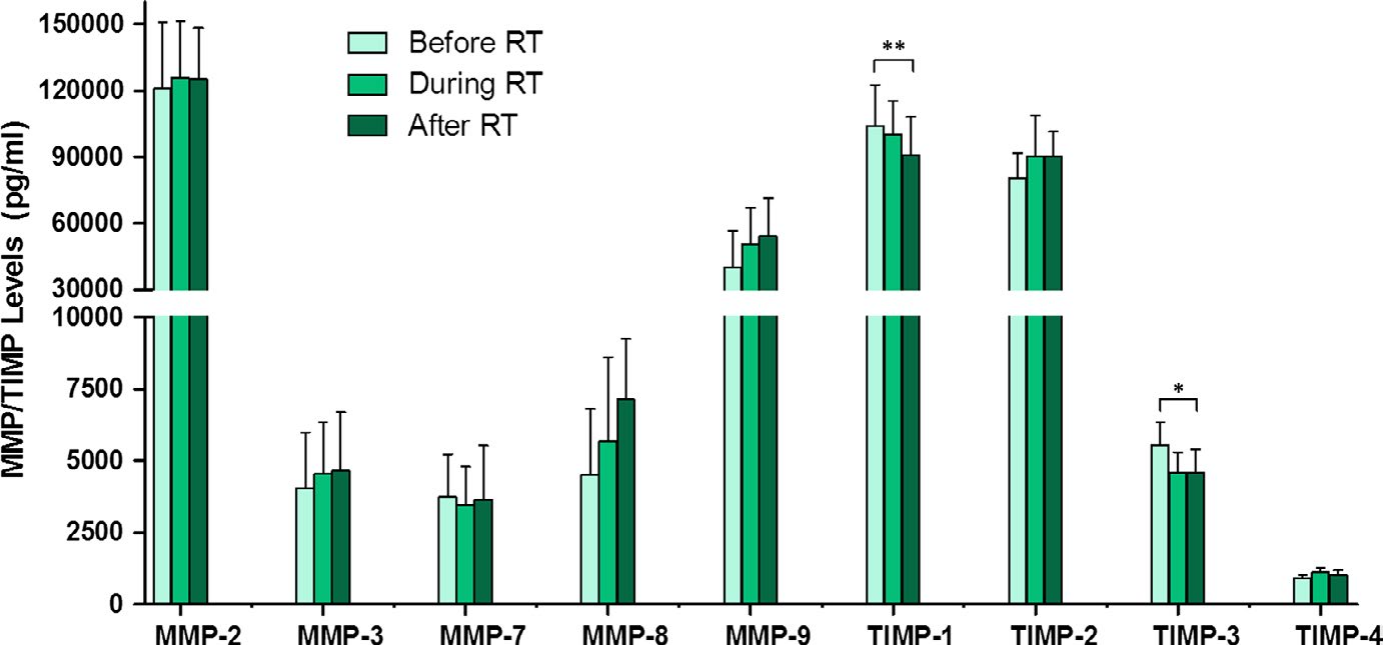
Time course of serum levels of MMPs and TIMPs before, during and after RT. Values are presented as median ± SD (error bars); * *P* < .05 and ** *P* < .01

As described in the Material and Methods section, ELISA‐like fluorescence immunoassay was used for the determination and quantification of serum levels of MMPs and TIMPs. MMP‐2, ‐3, ‐7, ‐8, ‐9, TIMP‐1, ‐2, ‐3 and ‐4 were detected, and Figure 1 shows the time course of their serum levels plotted against RT (before, during and after treatment). These results demonstrate a slight increase in the levels of most MMP and TIMP with RT. However, only the levels of TIMP‐1 and TIMP‐3, which decreased with RT, were statistically significant. Regardless of treatment, it is worth noting that MMP‐2, ‐9, TIMP‐1 and ‐2 serum levels were much higher than in the rest of the proteins analysed. In general terms, MMPs and TIMPs showed a tendency to increase, with the exception of TIMP‐1 and TIMP‐3 where the opposite was observed.

Due to the small sample size of our study, we searched different databases to identify works that support our pilot results. No studies showed similar results regarding the correlation between MMP and TIMP serum levels and RT. Only the study from the GEO database (GSE101920) was similar to our own. However, in that study, only 5 patients had the pre‐ and post‐RT biopsy samples matched. Despite this limitation, pairs of samples were analysed but no significant results were obtained (Figure S1).

### 3.2 MMP and TIMP serum level correlation

The correlation between serum levels of MMPs and TIMPs before, during and after RT was obtained using the Spearman correlation coefficient, *ρ* (rho). According to the rho values, when the association is positive, 0 < *ρ* < 1, the expression level of the two genes compared shows a similar trend, either an increase or decrease in their expression level. A negative association, ‐1 < *ρ* < 0, means that the expression levels of the two genes compared are opposite. Finally, there is no linear correlation between the genes studied when *ρ* = 0.

**Figure 2:**
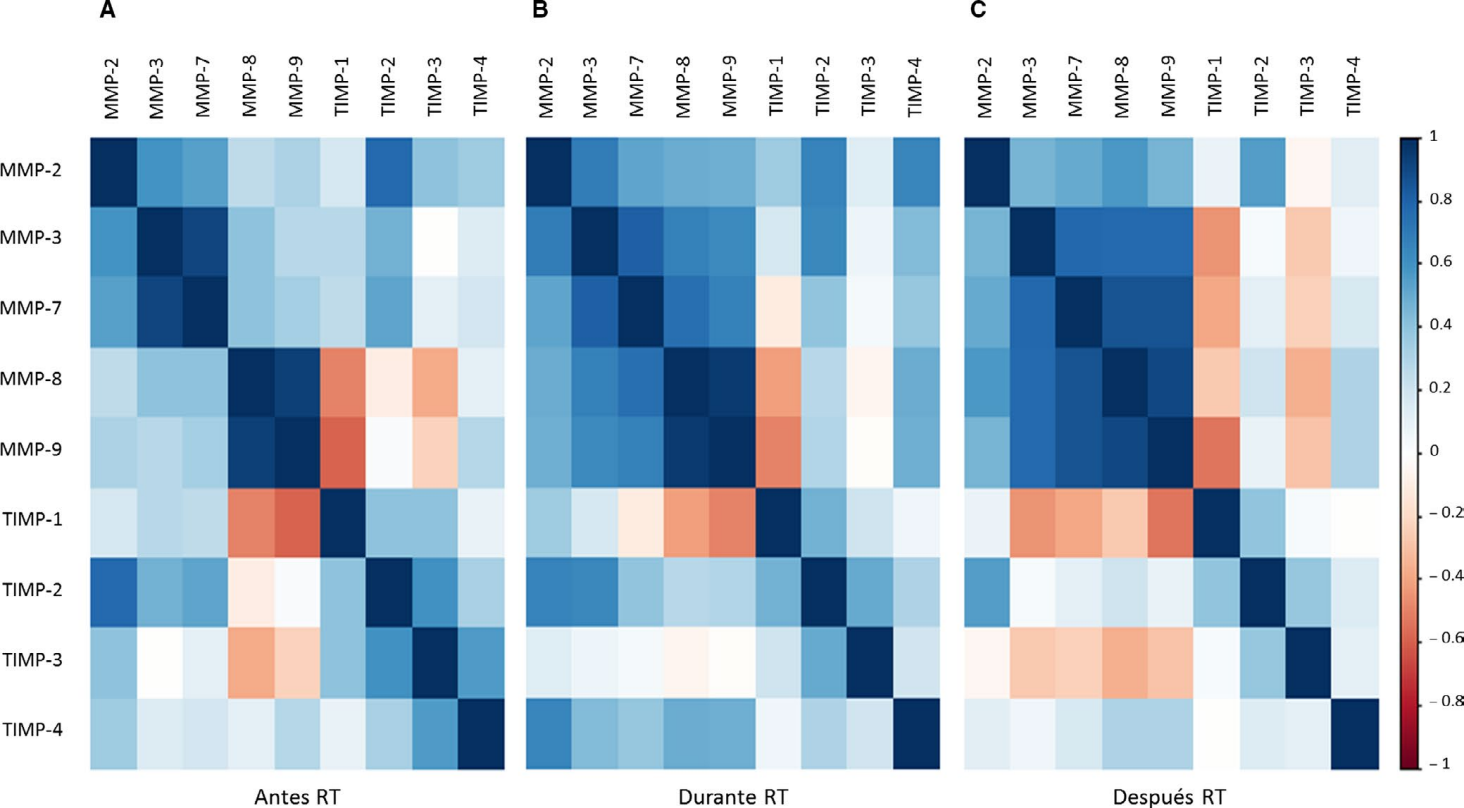
Correlation between the serum levels of MMPs and TIMPs before (A), during (B) and after (C) RT. The range of colours represents the different values of rho (*ρ*): positive correlation (0 < *ρ* < 1), negative correlation (−1 < *ρ* < 0) and no correlation (*ρ* = 0)

Figure 2 shows the correlation between the levels of MMPs and TIMPs before (Figure 2A), during (Figure 2B) and after (Figure 2C) RT. A positive correlation was found between the levels of MMPs at all times over the treatment, being this correlation stronger with RT. Nevertheless, positive and negative correlations have been found as a function of patient treatment time, with an increase in negative correlations for TIMP‐1 and ‐3 with RT.

### 3.3 MMP and TIMP serum levels by variables

The correlation between serum levels of all MMPs and TIMPs detected and the different variables was investigated, including patient‐dependent variables (age and menopausal status), tumour biology‐dependent variables (classification based on hormones, differentiation grade, positive or negative E‐cadherin and p53, Ki67 percentage and sentinel lymph node involvement) and RT‐related variables (RT regimen, lymph node RT and presence of radiotoxicity). Due to elevated number of graphs obtained after analysing all MMPs and TIMPs with these variables, only the statistically significant correlations are shown and discussed. The data corresponding to the protein levels according to all the variables can be seen in Tables S1‐S11.

**Figure 3:**
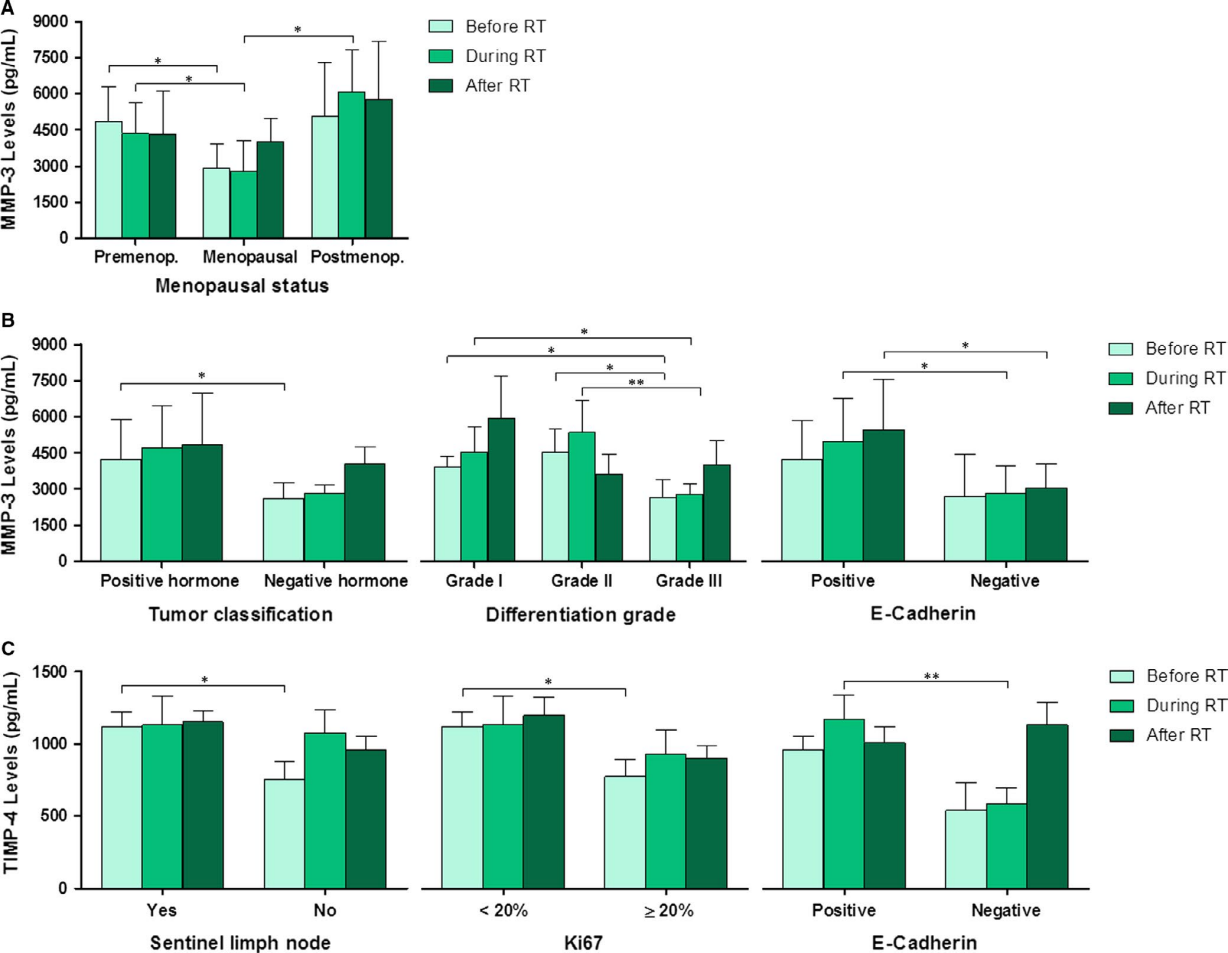
Serum levels of MMPs and TIMPs before, during and after RT in relation to patient‐dependent variables (A) and tumour biology‐dependent variables (B and C). Only the statistically significant variables are represented. (A) MMP‐3 levels in relation to menopausal status of the patients. (B) MMP‐3 levels in relation to tumour classification, differentiation grade and E‐cadherin presence. (C) TIMP‐4 levels in relation to sentinel lymph node, Ki67 percentage and E‐cadherin presence. Values are presented as median ± SD (error bars); * *P* < .05 and ** *P* < .01

Figure 3 shows the statistically significant correlations between serum levels of MMPs and TIMPs before, during and after RT and the patient‐dependent variables (Figure 3A) and tumour biology‐dependent variables (Figure 3B and 3). The results showed that only MMP‐3 and TIMP‐4 levels were statistically significant for some variables. Figure 3A shows the correlation between MMP‐3 levels and menopausal status of the patient. In general, this protein levels were higher in post‐menopausal patients, but it is worth noting that for each group of patients (pre‐menopausal and post‐menopausal), the levels are higher at a different time of the treatment. Figure 3B shows the correlation between MMP‐3 levels and tumour classification, differentiation grade and E‐cadherin presence. MMP‐3 levels increased in patients with hormone‐positive tumours compared with those with hormone‐negative tumours, having an increase in both groups throughout the treatment with RT. Considering tumour differentiation degree, MMP‐3 levels were higher after RT in grade I and III tumours. However, in grade II tumours, protein levels were higher during treatment. Respect to E‐cadherin, patients with E‐cadherin positive tumours also showed higher MMP‐3 levels than those with negative E‐cadherin, having a progressive increase in the E‐cadherin positive group throughout RT. Figure 3C shows the correlation between TIMP‐4 levels and involvement of sentinel lymph node, Ki67 percentage and E‐cadherin presence. TIMP‐4 levels were similar in both groups of patients, independently of the involvement of sentinel lymph node, but it should be noted the low levels of this inhibitor before RT in the group with no sentinel lymph node involvement. Considering Ki67 protein, TIMP‐4 levels varied depending on the Ki67 percentage in the tumour, with higher expression in patients with Ki67 percentage score <20%. In patients with Ki67 ≥ 20%, TIMP‐4 levels increased during RT. Finally, TIMP‐4 levels were higher for E‐cadherin positive tumours; but it is noteworthy the large increase in TIMP‐4 in E‐cadherin negative tumours after RT. These results suggest the involvement of this inhibitor in tumour proliferation and invasion.

**Figure 4:**
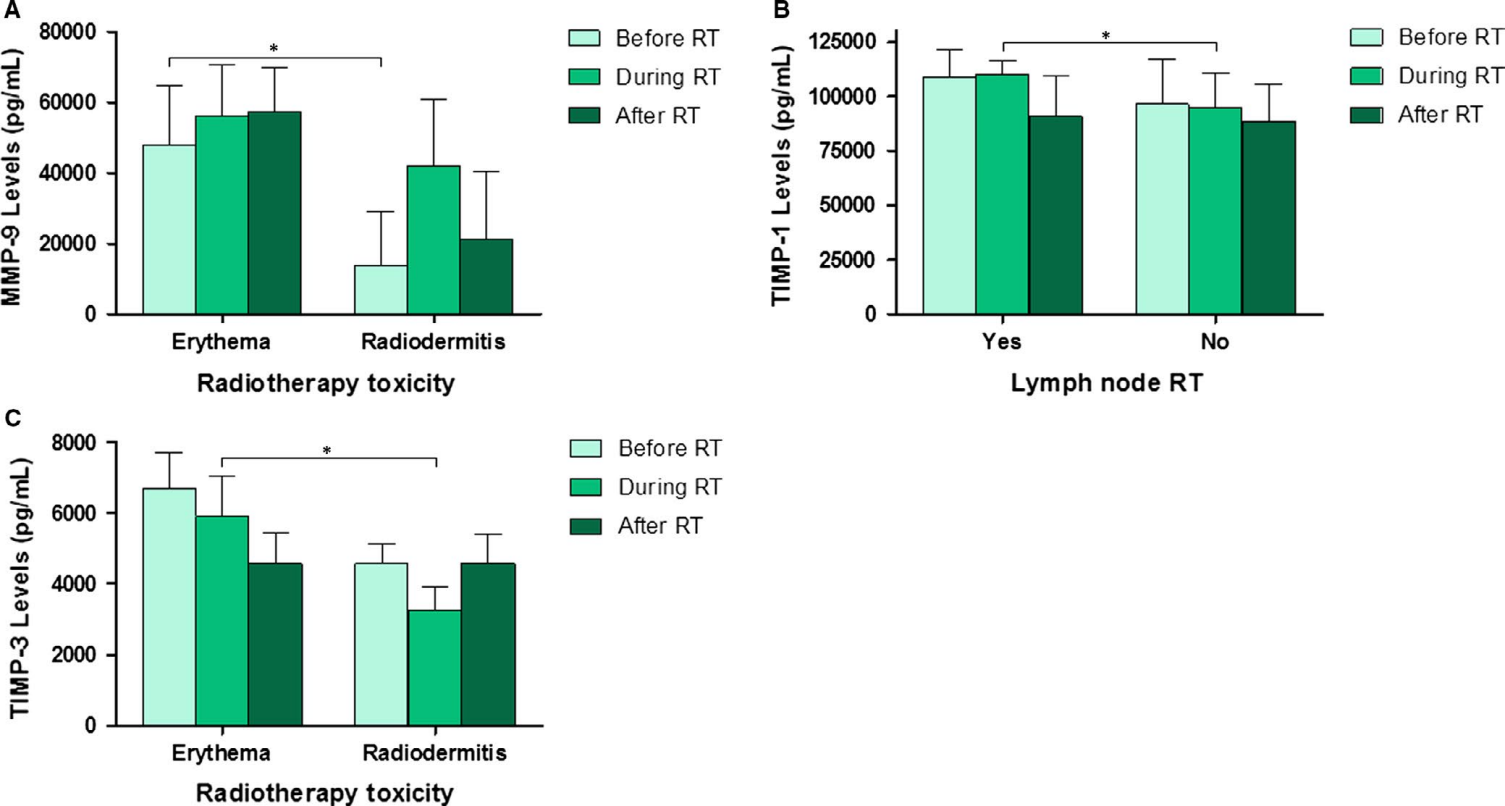
Serum levels of MMPs and TIMPs before, during and after RT based on RT‐related variables (A, B and C). Only the statistically significant variables are represented. (A) MMP‐9 levels in relation to radiation toxicity. (B) TIMP‐1 levels in relation to lymph node radiotherapy. (C) TIMP‐3 levels in relation to radiation toxicity. Values are presented as median ± SD (error bars); * *P* < .05

Figure 4 shows the statistically significant serum levels of MMPs and TIMPs before, during and after RT based on RT‐related variables. Regarding the RT‐related variables, Figure 4 shows that only MMP‐9, TIMP‐1 and ‐3 levels were found to be statistically significant for some of the variables. Figure 4A and 4 show a statistically significant correlation between MMP‐9 and TIMP‐3 levels with the type of radiation toxicity. MMP‐9 levels were much higher in patients with erythema, showing this group a slight increase throughout the treatment. Moreover, MMP‐9 levels in patients with radiodermitis were much higher during RT than before or after RT. However, TIMP‐3 levels were very similar in both groups of toxicity but levels decreased throughout treatment. Figure 4C shows a statistically significant correlation between TIMP‐1 levels and lymph node RT. TIMP‐1 levels were very similar in patients who have received lymph node RT to those who have not received it, with decreased levels in both groups at the end of RT.

### 3.4 Comparison of MMP and TIMP serum levels, according to tumour recurrence

Breast cancer recurrence was determined six months after the termination of RT. Overall survival was 100%, and disease‐free survival was 85% (recurrence in 3 patients) (Table 1). The data corresponding to the protein levels according to the tumour recurrence can be seen in Table S12.

**Figure 5:**
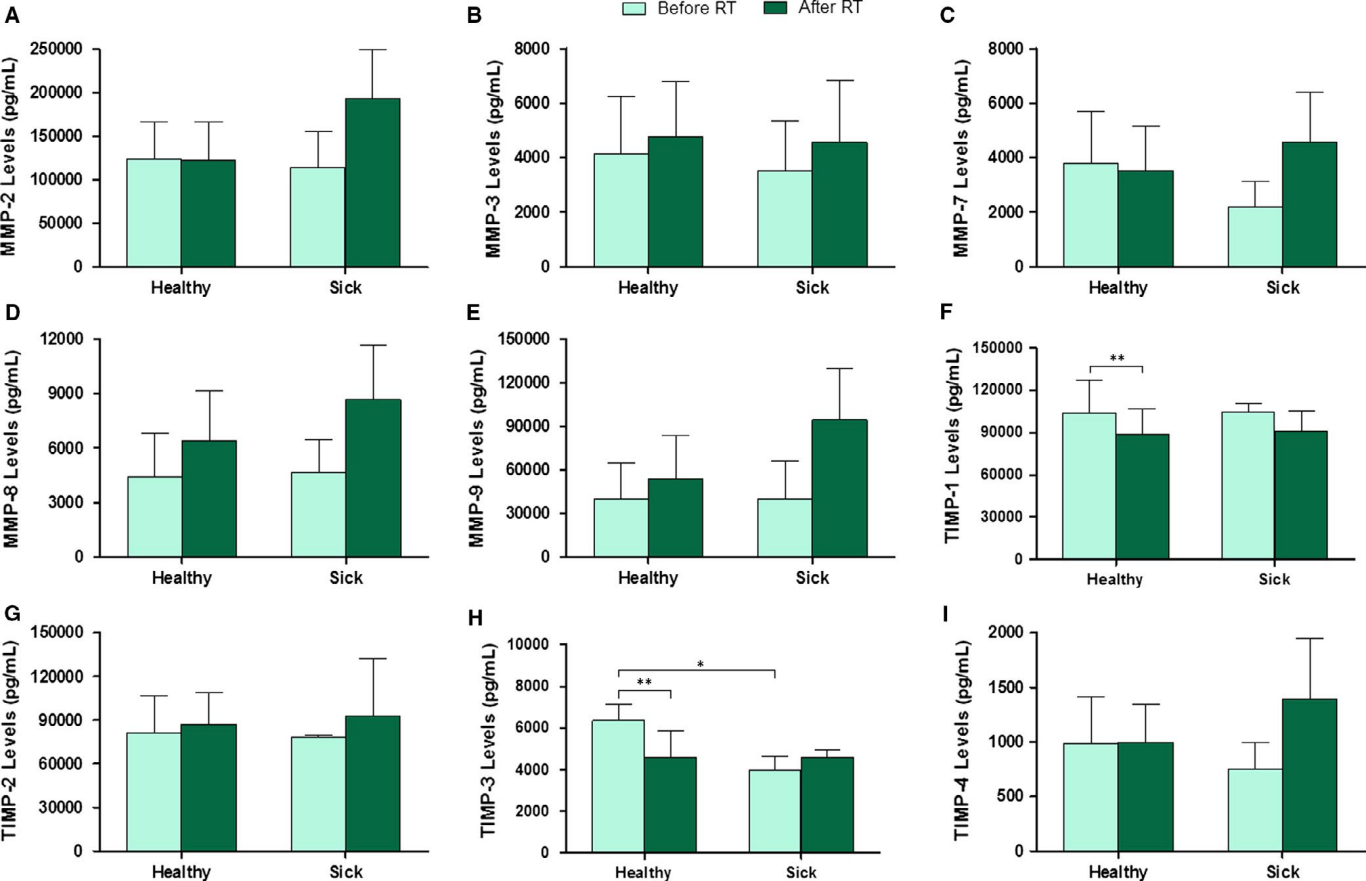
Serum levels of MMPs and TIMPs before and after RT in relation to the six‐month recurrence. Values are presented as median ± SD (error bars); * *P* < .05 and ** *P* < .01

The serum levels of all the MMPs and TIMPs detected were compared before and after RT, this time taking into account the recurrence variable (Figure 5). Patients were grouped into healthy (no recurrent BC) and sick (recurrent BC). Statistically significant values were found only for TIMP‐1 and ‐3 levels. TIMP‐1 levels (Figure 5F) were statistically significant when comparing the levels before and after RT in healthy patients. However, TIMP‐3 levels (Figure 5H) were statistically significant not only when comparing before and after RT in healthy patients, but also when comparing the levels between healthy and sick patients before RT.

## 4 DISCUSSION

This pilot study investigates the alterations in MMPs and TIMPs related to RT in BC patients. To our knowledge, this is the first work that examines the association between levels of such a wide range of MMPs and TIMPs and RT in BC patients.

RT is a highly targeted and effective treatment modality for BC. Successful RT in eradicating a tumour depends principally on the total radiation dose given, but the tolerance of the normal tissues surrounding the tumour limits this dose. There is significant variation between patients in the severity of toxicity following a given dose of RT. As mentioned above, side effects can be classified into acute and chronic (long‐term) and skin toxicity is the most common adverse effect in patients with BC.^35^

Little research has documented the effect of RT on MMPs and their tissue inhibitor expression in patients. Some authors have found that MMP‐9 serum levels before radiation were significantly higher compared with those obtained after RT, which suggests their usefulness as indicators of RT efficacy in patients with lung cancer.^36^ In contrast, our results show a slight increase in serum levels of most MMPs analysed after RT in patients with BC, although this increase was not statistically significant. RT also influences TIMP levels. Our findings revealed a statistically significant decrease in TIMP‐1 and ‐3 serum levels with RT (Figure 1). This could be associated with the increase of some MMPs analysed in this work. In this sense, it is important to consider that TIMPs are not only involved in MMP inhibition but also in different signalling pathways. Some authors have described that TIMP‐1 stimulates cancer invasion by inhibiting apoptosis, promoting tumour cell growth and regulating angiogenesis in metastatic BC.^37^, ^38^ A relationship between high serum levels of TIMP‐1 and poor prognosis in patients with BC has also been reported.^39^ Some studies have documented a correlation between low TIMP‐3 expression levels and an aggressive phenotype of BC and poor relapse‐free survival.^40^, ^41^ De Schutter et al studied a group of 46 head and neck squamous cell carcinoma patients treated with RT only and found that epigenetic silencing of TIMP‐3 was a predictor of better outcome.^42^

We are aware that the main limitation of this study is the small sample size. To mitigate this limitation, we have searched different database for similar results. Only the work by Tanic et al (GEO database) in biopsies from non‐inflammatory locally advanced patients with BC reported no changes in MMP or TIMP levels between the pre‐ (n = 5) and post‐RT (n = 5) group (Figure S1). The few pre‐ and post‐RT–matched sample number could explain the lack of statistically significant results related to MMP and TIMP level changes induced by RT. On the other hand, these data show differential MMP‐14 gene expression between responders (n = 30) and non‐responders (n = 12) to RT, being this gene down‐regulated in radiosensitive tumours in the preoperative setting.^34^

In our study, considering the *ρ* coefficient, a positive correlation was found between the serum levels of MMPs before, during and after RT as shown in Figure 2A, 2 and 2, respectively. It is worth noting that the correlation between genes becomes stronger with RT. Nevertheless, positive and negative correlations have been found as a function of patient treatment status, with an increase in negative correlations for TIMP‐1 and ‐3 with RT (Figure 2).

Our results show a correlation between MMP‐3 levels and menopausal status, tumour classification, differentiation degree and E‐cadherin presence (Figure 3). Several parameters have been investigated as prognostic predictors of BC, such as lymph node status, tumour size, histologic type, tumour grade, hormonal receptor status, ploidy and proliferating markers.^43^, ^44^ Some authors have suggested a key role of E‐cadherin in tumour development and growth within the lymph nodes.^45^ Matrix‐degrading enzymes have been related to BC progression,^46^ tumour vascularization, invasion and metastasis, differentiation, proliferation and apoptosis,^47^ and hence MMPs are now considered to be multifaceted during cancer progression. Recent evidence indicates that the same MMP may play an opposite role at different stages of cancer progression depending on the cancer type.^48^, ^49^

Tissue inhibitors are endogenous inhibitors of MMPs. These proteins have important roles as regulators of the activities of MMPs. The significance of TIMPs as both proteinase inhibitors and signalling molecules in their own right has also been described, as well as their role in the induction of proliferation and the inhibition of apoptosis.^50^

Most studies have focused on TIMP‐1, ‐2 and ‐3, and the relationship between high levels of TIMP‐1 and poor BC prognosis has also been confirmed.^51^ Nevertheless, little is known about TIMP‐4.

Analysing the effect of different treatment approaches on TIMP‐1 and MMP‐9 levels for late stage BC, Yuan et al found that after radio‐chemotherapy all patients showed lower MMP‐9 serum levels and higher TIMP‐1 levels than those before treatment.^52^ Other authors have found a correlation between low levels of TIMP‐3 and tumour aggressiveness (high tumour grading and lymph node metastasis) and poor disease‐free survival.^40^, ^41^

Our results suggest that MMP‐9 and TIMP‐3 levels could be predictive of RT toxicity, particularly, of acute effects such as erythema and radiodermitis. Some authors have described MMP levels alterations in cancer following single and fractionated radiation in vivo. Some authors found statistically increased levels of MMP‐2, ‐3, ‐9 and ‐14 in the colon of rats irradiated with a single dose of 10 Gy. MMP‐2 has usually been involved in gastrointestinal toxicity after irradiation in both preclinical 53 and clinical studies.^54^ These studies highlight possible differences in MMP and TIMP levels related to sample (tissue or serum), time since last dose and single vs fractionated radiation regimens.

Matrix metalloproteases and TIMP serum level changes are associated with normal cells being adversely affected by RT (Figure 4). We should consider that these MMPs are produced by both tumour and normal cells. RT could have promoted an increase in protein levels in cells from healthy tissue located in the treatment area, and therefore, they would also be involved in the response to RT, particularly in the acute response. The involvement of MMPs in the occurrence of late manifestations of RT such as radiation‐induced fibrosis cannot be ruled out. This has not been demonstrated in our work because of the short follow‐up period. Fibroblasts play the central role in wound healing through deposition and remodelling of collagen fibres. In irradiated tissue, fibroblasts have been shown to generate a disorganized deposition of collagen bundles. One likely mechanism resulting in disorganized collagen deposition is dysregulation of MMPs and TIMPs. These enzymes regulate extracellular matrix synthesis.^55^

After analysing the time course of TIMP serum levels with RT, our results showed a statistically significant correlation between TIMP‐1 and lymph node RT with increased levels during RT in comparison with BC patients without lymph node RT (Figure 4). We have also found that TIMP‐4 levels differ as a function of sentinel node involvement, Ki67 percentage and E‐cadherin protein presence (Figure 3), which suggest a potential use of these TIMPs as biomarkers of prognosis and response to RT. A tumour is clinically radioresistant when irradiation in unable to reduce its volume or when a recurrence takes place after a possible regression. Thus, it would be of great interest to identify biomarkers predictive of response to RT. In this sense, our results show higher levels of most of proteins analysed after RT in patients with recurrence, although these differences also were not statistically significant (Figure 5), suggesting their role in the prediction of clinical outcome in RT treated patients.

To our knowledge, no studies have assessed the determination of such a wide range of MMPs and TIMPs in serum of patients with BC before, during and after RT. Clearly, MMP and TIMP levels could be influenced by many factors, including sample (serum or tissue) and RT regimen. This work has evaluated the imbalance between MMP and TIMP levels induced by RT. Although our study is limited by the small sample size, these preliminary evidences aim to do additional studies for further confirmation of our results.

## CONFLICT OF INTEREST

The authors confirm that there are no conflicts of interest.

## AUTHORS’ CONTRIBUTION

MAO‐U, CG‐L, JAM and MIN: conceptualization; MAO‐U, MZ, SR‐A, JL and RdM: methodology; JAM and MIN: validation; FA‐C, JPA and ARG: formal analysis; MAO‐U and CG‐L: investigation; JAM and MIN: resources; MAO‐U and FA‐C: data curation; MAO‐U and MIN: writing (original, review and editing); MZ, RdM, JAM and MIN: supervision; JAM and MIN: funding acquisition.

## Data Availability

The data that support the findings of this study are available from the corresponding author upon reasonable request.
