# Supplementary figures and images for: Matrix metalloproteases and TIMPs as prognostic biomarkers in breast cancer patients treated with radiotherapy: A pilot study
Source: J Cell Mol Med. 2019 Sep 30;24(1):139–48. doi: 10.1111/jcmm.14671 (PMC6933337; doi:10.1111/jcmm.14671)

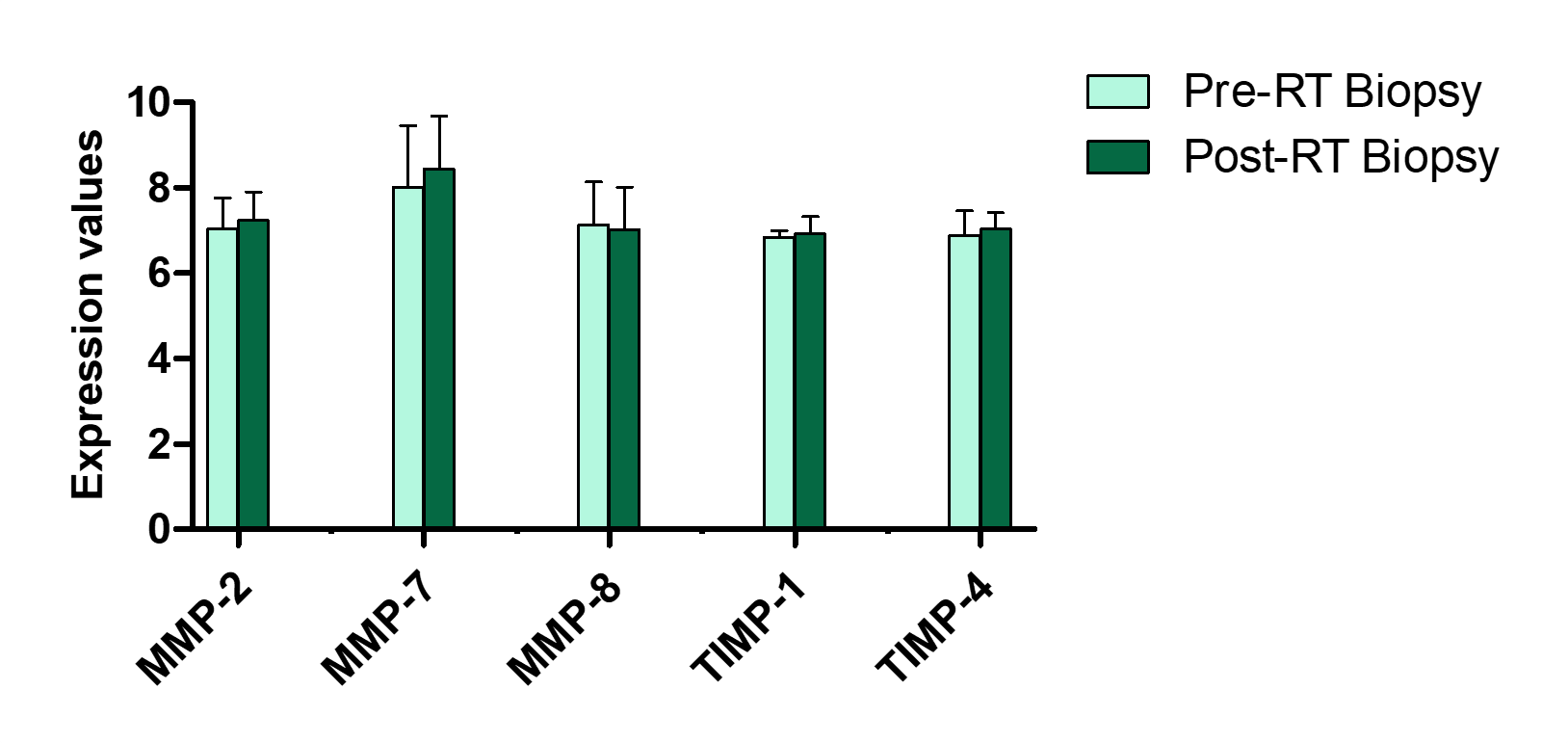

Supplement: Supplementary file 1 [file JCMM-24-139-s001.tif]
