# Supplementary material for: Matrix metalloproteases and TIMPs as prognostic biomarkers in breast cancer patients treated with radiotherapy: A pilot study
Source: J Cell Mol Med. 2019 Sep 30;24(1):139–48. doi: 10.1111/jcmm.14671 (PMC6933337; doi:10.1111/jcmm.14671)
Supplement: Supplementary file 2 [file JCMM-24-139-s002.docx]

**SUPPLEMENTARY TABLES**

**Table S1:** MMP and TIMP levels (pg/ml) according to age.

| Age | RT Times | | |
| --- | --- | --- | --- |
|  | **Before RT** | **During RT** | **After RT** |
| MMP-2 |  |  |  |
| ≤ 50 years | 121927.00 | 125922.20 | 124698.90 |
| > 50 years | 119687.70 | 134492.40 | 126213.90 |
| MMP-3 |  |  |  |
| ≤ 50 years | 4859.34 | 4356.31 | 4335.85 |
| > 50 years | 3841.18 | 4689.88 | 4648.65 |
| MMP-7 |  |  |  |
| ≤ 50 years | 3773.33 | 3296.55 | 3534.10 |
| > 50 years | 3630.06 | 3826.54 | 4038.44 |
| MMP-8 |  |  |  |
| ≤ 50 years | 5626.55 | 8494.93 | 5608.00 |
| > 50 years | 4026.62 | 4778.83 | 8012.33 |
| MMP-9 |  |  |  |
| ≤ 50 years | 39665.70 | 63125.46 | 54077.79 |
| > 50 years | 39951.88 | 44560.38 | 55773.34 |
| TIMP-1 |  |  |  |
| ≤ 50 years | 104022.90 | 95565.28 | 83648.65 |
| > 50 years | 104377.20 | 100503.20 | 93016.74 |
| TIMP-2 |  |  |  |
| ≤ 50 years | 78649.05 | 99017.58 | 83726.32 |
| > 50 years | 87248.23 | 83962.55 | 93323.47 |
| TIMP-3 |  |  |  |
| ≤ 50 years | 5068.02 | 5986.05 | 4583.22 |
| > 50 years | 5951.42 | 4583.22 | 4583.22 |
| TIMP-4 |  |  |  |
| ≤ 50 years | 958.49 | 1098.27 | 947.24 |
| > 50 years | 853.65 | 1109.37 | 1227.94 |

**Table S2:** MMP and TIMP levels (pg/ml) according to menopausal status.

| Menopausal  status | RT Times | | |
| --- | --- | --- | --- |
|  | **Before RT** | **During RT** | **After RT** |
| MMP-2 |  |  |  |
| Premenopausal | 121927.00 | 125922.20 | \| 124698,90 \| \| --- \| \|  \| \|  \| |
| Menopausal | 119687.70 | 131367.60 | 111510,80 |
| Postmenopausal | 136020.00 | 164032.80 | 158902,60 |
| MMP-3 |  |  |  |
| Premenopausal | \| 4859,34 \| \| --- \| \|  \| \|  \| | \| 4356,31 \| \| --- \| \|  \| \|  \| | \| 4335,85 \| \| --- \| \|  \| \|  \| |
| Menopausal | 2936,91 | 2806,75 | 4038,44 |
| Postmenopausal | 5066,38 | 6079,90 | 5757,67 |
| MMP-7 |  |  |  |
| Premenopausal | 3773.33 | 3296.55 | 3534.10 |
| Menopausal | 2668.38 | 2806.75 | 3419.47 |
| Postmenopausal | 5066.38 | 6079.90 | 5757.67 |
| MMP-8 |  |  |  |
| Premenopausal | 5626.55 | 8494.93 | 5608.00 |
| Menopausal | 3427.05 | 2897.48 | 5875.88 |
| Postmenopausal | 5049.31 | 6832.01 | 8707.82 |
| MMP-9 |  |  |  |
| Premenopausal | 39665.70 | 63125.46 | 54077.79 |
| Menopausal | 33855.59 | 33633.46 | 54680.54 |
| Postmenopausal | 43494.07 | 60910.10 | 67944.46 |
| TIMP-1 |  |  |  |
| Premenopausal | 104022.90 | 95565.28 | 83648.65 |
| Menopausal | 106008.00 | 105253.50 | 93016.74 |
| Postmenopausal | 101277.20 | 99273.33 | 99767.51 |
| TIMP-2 |  |  |  |
| Premenopausal | 78649.05 | 99017.58 | 83726.32 |
| Menopausal | 85684.75 | 82172.82 | 83509.07 |
| Postmenopausal | 105196.80 | 92709.03 | 103117.30 |
| TIMP-3 |  |  |  |
| Premenopausal | 5068.02 | 5986.05 | 4583.22 |
| Menopausal | 5951.42 | 4583.22 | 4583.22 |
| Postmenopausal | 5811.94 | 4583.22 | 5068.02 |
| TIMP-4 |  |  |  |
| Premenopausal | 958.49 | 1098.27 | 947.24 |
| Menopausal | 754.79 | 957.07 | 995.00 |
| Postmenopausal | 1146.02 | 1316.53 | 1414.29 |

**Table S3:** MMP and TIMP levels (pg/ml) according to tumor classification.

| Tumor  classification | RT Times | | |
| --- | --- | --- | --- |
|  | **Before RT** | **During RT** | **After RT** |
| MMP-2 |  |  |  |
| Hormone-negative | 121023.20 | 122170.00 | 123582.20 |
| Hormone-positive | 135030.90 | 167337.60 | 208103.30 |
| MMP-3 |  |  |  |
| Hormone-negative | 4209.32 | 4689.88 | 4836.71 |
| Hormone-positive | 2598.25 | 2806.75 | 4038.44 |
| MMP-7 |  |  |  |
| Hormone-negative | 3773.33 | 3682.47 | 3534.10 |
| Hormone-positive | 2634.46 | 2806.75 | 4038.44 |
| MMP-8 |  |  |  |
| Hormone-negative | 4761.10 | 5994.53 | 6397.28 |
| Hormone-positive | 3427.05 | 2897.48 | 8900.79 |
| MMP-9 |  |  |  |
| Hormone-negative | 40179.44 | 51022.85 | 54077.79 |
| Hormone-positive | 34070.35 | 33633.46 | 75819.80 |
| TIMP-1 |  |  |  |
| Hormone-negative | 104022.90 | 100503.20 | 89774.15 |
| Hormone-positive | 102973.00 | 105784.20 | 95912.63 |
| TIMP-2 |  |  |  |
| Hormone-negative | 80219.73 | 95289.55 | 83726.32 |
| Hormone-positive | 91211.77 | 82172.82 | 102631.20 |
| TIMP-3 |  |  |  |
| Hormone-negative | 5552.81 | 4839.71 | 4583.22 |
| Hormone-positive | 5466.62 | 3920.76 | 4583.23 |
| TIMP-4 |  |  |  |
| Hormone-negative | 958.49 | 1131.20 | 1006.67 |
| Hormone-positive | 540.76 | 664.16 | 1146.02 |

**Table S4:** MMP and TIMP levels (pg/ml) according to differentiation grade.

| Differenctiation  grade | RT Times | | |
| --- | --- | --- | --- |
|  | **Before RT** | **During RT** | **After RT** |
| MMP-2 |  |  |  |
| Grade I | 126454.00 | 124752.70 | 139389.60 |
| Grade II | 96235.00 | 113071.10 | 123050.00 |
| Grade III | 124862.60 | 144320.20 | 147288.60 |
| MMP-3 |  |  |  |
| Grade I | 3953.66 | 4553.57 | 5973.71 |
| Grade II | 4553.57 | 5378.37 | 3633.61 |
| Grade III | 2668.38 | 2806.75 | 4038.44 |
| MMP-7 |  |  |  |
| Grade I | 3953.66 | 4553.57 | 4929.69 |
| Grade II | 3804.65 | 3705.69 | 3315.63 |
| Grade III | 2668.38 | 2806.75 | 4038.44 |
| MMP-8 |  |  |  |
| Grade I | 2868.56 | 5473.22 | 7861.81 |
| Grade II | 6780.16 | 7587.50 | 6397.28 |
| Grade III | 3427.05 | 2897.48 | 7169.04 |
| MMP-9 |  |  |  |
| Grade I | 39737.12 | 46928.70 | 54071.31 |
| Grade II | 40621.76 | 60719.96 | 54239.75 |
| Grade III | 34070.35 | 33633.46 | 75819.80 |
| TIMP-1 |  |  |  |
| Grade I | 107557.90 | 97607.49 | 90907.00 |
| Grade II | 104305.70 | 109028.40 | 88865.94 |
| Grade III | 99433.27 | 103170.70 | 92998.00 |
| TIMP-2 |  |  |  |
| Grade I | 81163.03 | 97778.56 | 101435.90 |
| Grade II | 71214.01 | 80436.14 | 72625.42 |
| Grade III | 87324.83 | 82172.82 | 90117.76 |
| TIMP-3 |  |  |  |
| Grade I | 7657.20 | 7040.66 | 4583.22 |
| Grade II | 4583.22 | 4583.22 | 3988.30 |
| Grade III | 5466.62 | 4583.22 | 4583.22 |
| TIMP-4 |  |  |  |
| Grade I | 1002.98 | 1113.01 | 1307.76 |
| Grade II | 842.49 | 1192.83 | 898.89 |
| Grade III | 540.76 | 664.16 | 958.31 |

**Table S5:** MMP and TIMP levels (pg/ml) according to E-cadherin.

| E-cadherin | RT Times | | |
| --- | --- | --- | --- |
|  | **Before RT** | **During RT** | **After RT** |
| MMP-2 |  |  |  |
| Positive | 121023.20 | 125922.20 | 125231.00 |
| Negative | 135030.90 | 116706.40 | 137599.00 |
| MMP-3 |  |  |  |
| Positive | 4209.32 | 4965.97 | 5451.70 |
| Negative | 2668.38 | 2806.75 | 3038.18 |
| MMP-7 |  |  |  |
| Positive | 3773.33 | 3849.76 | 4242.87 |
| Negative | 2668.38 | 2806.75 | 3038.18 |
| MMP-8 |  |  |  |
| Positive | 4761.10 | 6750.02 | 6397.28 |
| Negative | 3427.05 | 2897.48 | 8012.33 |
| MMP-9 |  |  |  |
| Positive | 40179.44 | 55633.09 | 54077.79 |
| Negative | 34070.35 | 33633.46 | 69478.04 |
| TIMP-1 |  |  |  |
| Positive | 105931.80 | 103266.50 | 89688.48 |
| Negative | 94045.02 | 99338.10 | 92998.00 |
| TIMP-2 |  |  |  |
| Positive | 80219.73 | 101380.20 | 83726.32 |
| Negative | 91211.77 | 77734.42 | 93323.47 |
| TIMP-3 |  |  |  |
| Positive | 5552.81 | 5901.19 | 4583.22 |
| Negative | 5466.62 | 4583.22 | 4583.22 |
| TIMP-4 |  |  |  |
| Positive | 958.49 | 1171.10 | 1006.67 |
| Negative | 540.76 | 587.22 | 1131.82 |

**Table S6:** MMP and TIMP levels (pg/ml) according to p53.

| p53 | RT Times | | |
| --- | --- | --- | --- |
|  | **Before RT** | **During RT** | **After RT** |
| MMP-2 |  |  |  |
| Positive | 134996.00 | 173126.40 | 205585.00 |
| Negative | 117400.10 | 119587.40 | 124114.30 |
| MMP-3 |  |  |  |
| Positive | 3742.02 | 3296.55 | 6771.62 |
| Negative | 4159.04 | 4553.57 | 3742.02 |
| MMP-7 |  |  |  |
| Positive | 3742.02 | 3296.55 | 6771.62 |
| Negative | 3736.80 | 3659.25 | 3326.18 |
| MMP-8 |  |  |  |
| Positive | 3188.36 | 5473.22 | 7940.81 |
| Negative | 4647.72 | 5912.54 | 6397.28 |
| MMP-9 |  |  |  |
| Positive | 40166.64 | 42192.06 | 94332.68 |
| Negative | 39737.12 | 50546.22 | 54084.27 |
| TIMP-1 |  |  |  |
| Positive | 103740.10 | 105934.00 | 90869.53 |
| Negative | 104305.70 | 100407.40 | 90682.37 |
| TIMP-2 |  |  |  |
| Positive | 96671.81 | 113049.10 | 93088.37 |
| Negative | 79334.64 | 87488.96 | 80305.48 |
| TIMP-3 |  |  |  |
| Positive | 6350.03 | 7040.66 | 5552.81 |
| Negative | 5552.81 | 4583.22 | 4583.22 |
| TIMP-4 |  |  |  |
| Positive | 898.89 | 1047.17 | 1017.74 |
| Negative | 928.78 | 1113.01 | 995.59 |

**Table S7:** MMP and TIMP levels (pg/ml) according to the Ki67.

| Ki67 | RT Times | | |
| --- | --- | --- | --- |
|  | **Before RT** | **During RT** | **After RT** |
| MMP-2 |  |  |  |
| < 20% | 116225.00 | 119454.00 | 123582.20 |
| ≥ 20% | 145164.40 | 167337.60 | 153418.70 |
| MMP-3 |  |  |  |
| < 20% | 4209.32 | 4689.88 | 4836.71 |
| ≥ 20% | 3401.24 | 3178.51 | 4093.59 |
| MMP-7 |  |  |  |
| < 20% | 3879.16 | 3849.76 | 3534.10 |
| ≥ 20% | 2668.38 | 2806.75 | 4038.44 |
| MMP-8 |  |  |  |
| < 20% | 4761.10 | 5994.53 | 5608.00 |
| ≥ 20% | 3427.05 | 4411.05 | 8302.85 |
| MMP-9 |  |  |  |
| < 20% | 43442.98 | 51022.85 | 54077.79 |
| ≥ 20% | 25782.84 | 33633.46 | 59092.17 |
| TIMP-1 |  |  |  |
| < 20% | 104377.20 | 97777.56 | 88780.27 |
| ≥ 20% | 104022.90 | 107481.20 | 92998.00 |
| TIMP-2 |  |  |  |
| < 20% | 80219.73 | 86763.62 | 86932.03 |
| ≥ 20% | 87324.83 | 96361.79 | 90117.76 |
| TIMP-3 |  |  |  |
| < 20% | 5552.81 | 4583.22 | 4583.22 |
| ≥ 20% | 5466.62 | 4839.71 | 4583.22 |
| TIMP-4 |  |  |  |
| < 20% | 1119.53 | 1131.20 | 1199.44 |
| ≥ 20% | 771.82 | 1131.20 | 898.89 |

**Table S8:** MMP and TIMP levels (pg/ml) according to sentinel lymph node.

| Sentinel  lymph node | RT Times | | |
| --- | --- | --- | --- |
|  | **Before RT** | **During RT** | **After RT** |
| MMP-2 |  |  |  |
| Yes | 125550.10 | 119454.00 | 133018.10 |
| No | 105482.10 | 167337.60 | 123582.20 |
| MMP-3 |  |  |  |
| Yes | 4106.63 | 4689.88 | 5451.70 |
| No | 3950.53 | 3925.06 | 4093.59 |
| MMP-7 |  |  |  |
| Yes | 3845.23 | 3826.54 | 3534.10 |
| No | 3401.24 | 3178.51 | 4038.44 |
| MMP-8 |  |  |  |
| Yes | 4517.61 | 5692.88 | 6340.26 |
| No | 4558.32 | 5468.19 | 7169.04 |
| MMP-9 |  |  |  |
| Yes | 46315.30 | 50481.88 | 53062.73 |
| No | 25782.84 | 51456.01 | 59092.17 |
| TIMP-1 |  |  |  |
| Yes | 104377.20 | 96288.99 | 89886.47 |
| No | 104022.90 | 107481.20 | 90775.95 |
| TIMP-2 |  |  |  |
| Yes | 87277.34 | 95289.55 | 94575.37 |
| No | 74588.73 | 83962.55 | 80538.46 |
| TIMP-3 |  |  |  |
| Yes | 7274.75 | 5644.70 | 4583.22 |
| No | 4583.22 | 4583.22 | 4583.22 |
| TIMP-4 |  |  |  |
| Yes | 1119.53 | 1131.20 | 1151.68 |
| No | 754.79 | 1076.44 | 958.31 |

**Table S9:** MMP and TIMP levels (pg/ml) according to RT regimen.

| RT regimen | RT Times | | |
| --- | --- | --- | --- |
|  | **Before RT** | **During RT** | **After RT** |
| MMP-2 |  |  |  |
| Conventional | 117400.10 | 124752.70 | 139389.60 |
| Hypofractionated | 124646.30 | 127091.70 | 123050.00 |
| MMP-3 |  |  |  |
| Conventional | 3523.32 | 3296.55 | 4553.57 |
| Hypofractionated | 4553.57 | 5378.37 | 4743.73 |
| MMP-7 |  |  |  |
| Conventional | 3523.32 | 3296.55 | 3523.32 |
| Hypofractionated | 3804.65 | 3993.83 | 3742.02 |
| MMP-8 |  |  |  |
| Conventional | 3665.74 | 5473.22 | 7940.81 |
| Hypofractionated | 5450.90 | 7587.50 | 6397.28 |
| MMP-9 |  |  |  |
| Conventional | 40166.64 | 46928.70 | 54084.27 |
| Hypofractionated | 23591.63 | 60719.96 | 54239.75 |
| TIMP-1 |  |  |  |
| Conventional | 96474.91 | 97607.49 | 90869.53 |
| Hypofractionated | 109058.00 | 110099.50 | 90682.37 |
| TIMP-2 |  |  |  |
| Conventional | 81104.82 | 87488.96 | 93088.37 |
| Hypofractionated | 77963.46 | 105234.60 | 87147.15 |
| TIMP-3 |  |  |  |
| Conventional | 6350.03 | 4583.22 | 4583.22 |
| Hypofractionated | 5552.81 | 5096.20 | 4583.22 |
| TIMP-4 |  |  |  |
| Conventional | 898.89 | 868.86 | 1307.76 |
| Hypofractionated | 928.78 | 1192.83 | 898.89 |

**Table S10:** MMP and TIMP levels (pg/ml) according to lymph node RT.

| Lymph  node RT | RT Times | | |
| --- | --- | --- | --- |
|  | **Before RT** | **During RT** | **After RT** |
| MMP-2 |  |  |  |
| Yes | 124646.30 | 161548.80 | 124114.30 |
| No | 117400.10 | 119587.40 | 137722.90 |
| MMP-3 |  |  |  |
| Yes | 4259.60 | 4553.57 | 4553.57 |
| No | 3523.32 | 4159.04 | 4929.69 |
| MMP-7 |  |  |  |
| Yes | 3742.02 | 3705.69 | 3742.02 |
| No | 3523.32 | 3296.55 | 3326.18 |
| MMP-8 |  |  |  |
| Yes | 4387.50 | 4084.45 | 6397.28 |
| No | 4647.72 | 5912.54 | 7861.81 |
| MMP-9 |  |  |  |
| Yes | 27974.06 | 50417.53 | 57306.92 |
| No | 46264.21 | 50546.22 | 54071.31 |
| TIMP-1 |  |  |  |
| Yes | 109058.00 | 110099.50 | 90869.53 |
| No | 96474.91 | 94970.49 | 88865.94 |
| TIMP-2 |  |  |  |
| Yes | 77977.86 | 87488.96 | 93088.37 |
| No | 81104.82 | 92800.55 | 80305.48 |
| TIMP-3 |  |  |  |
| Yes | 5552.81 | 4583.22 | 4583.22 |
| No | 7040.66 | 4583.22 | 4583.22 |
| TIMP-4 |  |  |  |
| Yes | 842.49 | 1105.72 | 898.89 |
| No | 1002.98 | 1113.01 | 1307.76 |

**Table S11:** MMP and TIMP levels (pg/ml) according to RT toxicity.

| RT toxicity | RT Times | | |
| --- | --- | --- | --- |
|  | **Before RT** | **During RT** | **After RT** |
| MMP-2 |  |  |  |
| Erythema | 125550.10 | 122170.00 | 133018.10 |
| Radiodermitis | 96235.00 | 173126.40 | 123050.00 |
| MMP-3 |  |  |  |
| Erythema | 4106.63 | 5102.28 | 6372.67 |
| Radiodermitis | 4159.04 | 4553.57 | 3742.02 |
| MMP-7 |  |  |  |
| Erythema | 3739.41 | 3477.90 | 4127.93 |
| Radiodermitis | 3804.65 | 3705.69 | 3742.02 |
| MMP-8 |  |  |  |
| Erythema | 4761.10 | 7012.42 | 7972.83 |
| Radiodermitis | 3188.36 | 3348.88 | 2547.69 |
| MMP-9 |  |  |  |
| Erythema | 48224.74 | 56109.71 | 57480.84 |
| Radiodermitis | 13739.34 | 42192.06 | 21271.95 |
| TIMP-1 |  |  |  |
| Erythema | 104022.90 | 97784.76 | 86332.94 |
| Radiodermitis | 95126.47 | 100407.40 | 95126.47 |
| TIMP-2 |  |  |  |
| Erythema | 81133.93 | 105108.30 | 90352.86 |
| Radiodermitis | 71214.01 | 80436.14 | 93088.37 |
| TIMP-3 |  |  |  |
| Erythema | 6695.34 | 5901.19 | 4583.22 |
| Radiodermitis | 4583.22 | 3258.30 | 4583.22 |
| TIMP-4 |  |  |  |
| Erythema | 995.59 | 1131.20 | 1006.67 |
| Radiodermitis | 567.90 | 808.41 | 821.36 |

**Table S12:** MMP and TIMP levels (pg/ml) according to tumor recurrence.

| Tumor  recurrence | RT Times | |
| --- | --- | --- |
|  | **Before RT** | **After RT** |
| MMP-2 |  |  |
| Healthy | 114729.10 | 193690.90 |
| Sick | 124646.30 | 123050.00 |
| MMP-3 |  |  |
| Healthy | 3523.32 | 4553.57 |
| Sick | 4159.04 | 4743.73 |
| MMP-7 |  |  |
| Healthy | 2208.46 | 4553.57 |
| Sick | 3804.65 | 3523.32 |
| MMP-8 |  |  |
| Healthy | 4647.72 | 8664.90 |
| Sick | 4387.50 | 6397.28 |
| MMP-9 |  |  |
| Healthy | 40166.64 | 94332.68 |
| Sick | 39737.12 | 54071.31 |
| TIMP-1 |  |  |
| Healthy | 104305.70 | 90869.53 |
| Sick | 103740.10 | 88865.94 |
| TIMP-2 |  |  |
| Healthy | 77977.86 | 93088.37 |
| Sick | 81163.03 | 87147.15 |
| TIMP-3 |  |  |
| Healthy | 3988.30 | 4583.22 |
| Sick | 6350.03 | 4583.22 |
| TIMP-4 |  |  |
| Healthy | 988.20 | 995.59 |
| Sick | 747.37 | 1393.15 |
